# Development of a Pure Certified Reference Material of D-Mannitol

**DOI:** 10.3390/molecules28196794

**Published:** 2023-09-25

**Authors:** Weizhu Chen, Yiping Zhang, Hui Chen, Wenhui Jin, Xiaoying Chen, Xiaoyan Huang, Yanrou Xie, Hua Fang, Zhuan Hong

**Affiliations:** 1 Engineering Research Center of Marine Biological Resource Comprehensive Utilization, Third Institute of Oceanography, Ministry of Natural Resource, Xiamen 361005, China; wzchen@tio.org.cn (W.C.); chenhui@tio.org.cn (H.C.);; 2 Xiamen Ocean Vocational College, Xiamen 361100, China; 3 Fujian Provincial Key Laboratory of Island Conservation and Development, Island Research Center, MNR, Pingtan 350400, China; 4 College of Biology and Environment, Zhejiang Wanli University, Ningbo 315100, China

**Keywords:** D-mannitol, certified reference material, characterization, homogeneity study, stability monitoring, uncertainty evaluation

## Abstract

A new certified reference material (CRM) of D-mannitol (GBW(E) 100681) has been developed in this study. We describe the preparation, structure determination, characterization, homogeneity study, stability study, as well as uncertainty estimation. The main component was 99.91% ± 0.01%. The moisture content of the candidate CRM was 0.036% ± 0.002%, as measured by Karl Fischer titration. The nonvolatile and volatile impurities in the candidate CRM were all much less than 0.01%, which was determined by the ICP–MS and headspace GC–FID methods, respectively. The purity of the D-mannitol CRM was 99.9% ± 1.1% (*k* = 2), as measured by the two independent approaches involving the mass balance method (MB) and quantitative nuclear magnetic resonance technique (*q*NMR). The D-mannitol CRM was stable during the monitoring period for each temperature. It is stable for up to 48 months at room temperature and 28 days at 50 °C. The uncertainty was evaluated by combining the contributions from characterization, homogeneity, and stability. The developed D-mannitol CRM would effectively support method validation and proficiency testing, as well as effectively guarantee the accuracy, reliability, and comparability of results.

## 1. Introduction

D-Mannitol, chemically 1,2,3,4,5,6-hexanehexol (C_6_H_8_(OH)_6_), is the most abundant six-carbon sugar alcohol in nature. Due to its unique functional properties, such as low calorific value, no moisture absorption, high stability, and suitability as a sweetener, D-mannitol has broad application prospects in the chemical, food, and pharmaceutical industries [1,2]. However, D-mannitol also has some side effects including initial volume expansion (increasing the risk of heart failure), subsequent hypovolemia and hypotension, metabolic acidosis, and electrolyte imbalance, including hypernatremia and hypokalemia. Notwithstanding its clinical importance, high amounts of D-mannitol (1.4 g/kg body weight) may cause blood pressure change, mortality, or neurological outcomes [3,4]. To guarantee the routine monitoring of D-mannitol nationwide, a certified reference material (CRM) of D-mannitol is highly necessary and important. However, there is no D-mannitol CRM available currently. Thus, the development of a CRM for the determination of D-mannitol has become essentially indispensable.

A CRM is a substance or material that has one or more sufficiently homogeneous and stable property values with the known uncertainty for each property value at a certain confidence level. It is equipped with a certificate that describes the value of the specified property, the associated uncertainty, and a statement of metrological traceability [5]. It plays an important role by translating its accuracy to intended uses such as calibration, quality control, method validation, and the assignment of values to other materials [6].

To certify the CRM, a determination of purity must be carried out. Many techniques for purity assay can be used, such as MB and *q*NMR. MB method is an indirect method, in which the main component and all the detectable impurities (including impurities, volatile impurities, and moisture) of the CRM are comprehensively quantified. The purity is determined by subtracting all detected impurities from 100%. *q*NMR method is a direct method that is based on the direct proportionality of the signal response in the spectrum to the numbers of nuclei producing the corresponding resonance line. It does not require the determination of any impurity and only uses a primary CRM to determine the absolute content of the target compound [7,8]. It can overcome the shortcoming that the MB method might not detect any impurity. Therefore, it is usually used for the purity assay together with the MB method.

In this study, a novel D-mannitol CRM (GBW(E) 100681) was developed in accordance with ISO Guides and Chinese National Technical Specifications of Metrology JJF 1855-2020 [9,10,11]. The whole procedure, including structure analysis, stability, homogeneity, certified value, and corresponding uncertainty, is completely described. The certified value for the developed CRM was determined through MB and *q*NMR techniques. Using the MB approach, analysis of the principal component, moisture, nonvolatile impurities, and volatile impurities was performed separately through high-performance liquid chromatography with evaporative light scattering detector (HPLC–ELSD), Karl Fischer titration, inductively coupled plasma mass spectrometry (ICP–MS), and headspace gas chromatography using a flame-ionization detector (GC–FID), respectively. In *q*NMR measurement, benzoic acid CRM was used as an IS for direct assignment.

## 2. Results and Discussion

### 2.1. Structure Determination

**Figure 1:**
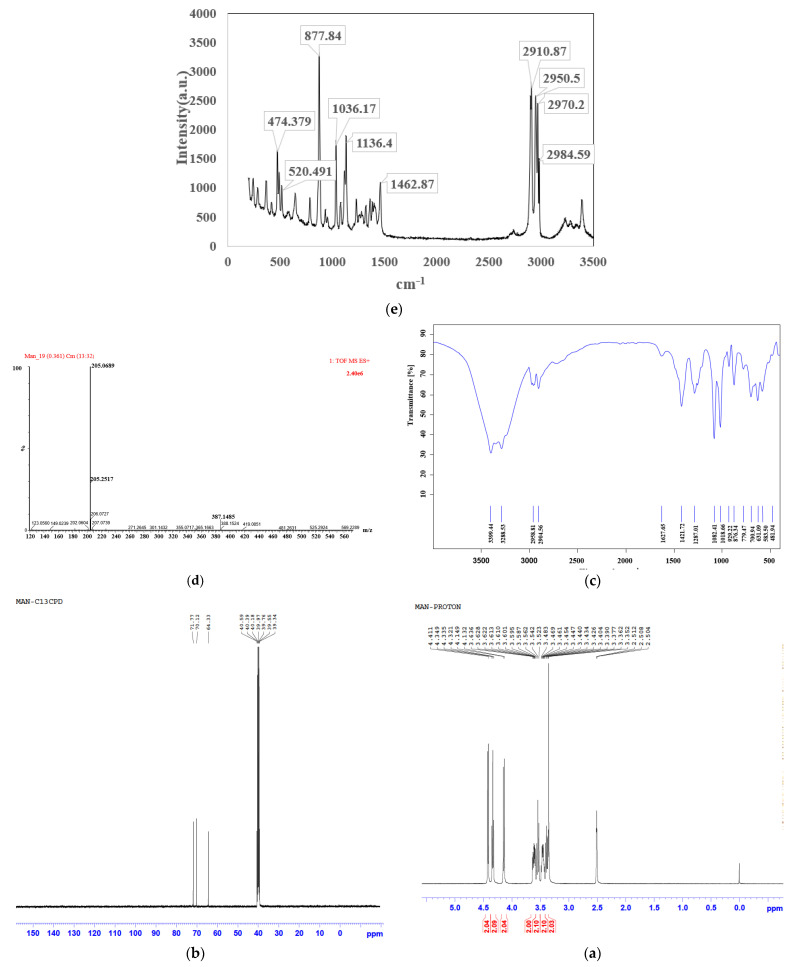
The spectrum of D-mannitol candidate CRM: (**a**) ^1^H NMR spectrum, (**b**) ^13^C NMR spectrum, (**c**) FT-IR spectrum, (**d**) HRMS, (**e**) Raman spectrum.

Structure determination aimed to verify the identity of the D-mannitol material. The ^1^H and ^13^C NMR spectra, IR spectrum, and HRMS for the candidate CRM were shown in Figure 1. The data of ^1^H and ^13^C NMR spectra are shown in Table S1, which are consistent with the chemical structure of D-mannitol (Figure S1). As can been seen from the IR spectrum (Figure 1c), the peak at 3400 cm^−1^ was ascribed to the O–H stretching vibration peak of D-mannitol. Bands at 2956 cm^−1^ and 2903 cm^−1^ were ascribed to C–H stretching vibration peaks in –CH and –CH_2_ of D-mannitol, respectively. The peaks at 1421 cm^−1^, 1289 cm^−1^, 1082 cm^−1^, and 630 cm^−1^ were ascribed to C–H deformation vibration modes in the –CH_2_, O–H deformation vibration peak in –CH_2_OH, O–H deformation vibration peak in –CHOH, and H deformation vibration peak in OH, respectively. For the HRMS spectrum, a strong peak at *m*/*z* 205.0689 was detected, which is consistent with the molecular ion [C_6_H_14_O_6_+Na]^+^ (calcd. for [C_6_H_14_O_6_+Na]^+^, 205.0688). This confirmed that the molecular formula was C_6_H_14_O_6_. The IR spectrum is in accordance with the corresponding D-mannitol standard IR spectrum from the SDBS database and the reported literature [12,13]. As can been seen from the Raman spectrum (Figure 1e), the result is also consistent with the SDBS database and the reported study [12,14]. And there is the peak of 1036 cm^−1^ in the Raman spectrum. It is indicated that D-mannitol is beta polymorph in the study [14]. The above results indicate that the candidate CRM is D-mannitol.

### 2.2. Development of the HPLC–ELSD Method

Several analytical methods have been reported for the determination of D-mannitol, including GC [15,16,17], high-performance liquid chromatography with electrochemical detection (HPLC-PAD) [18,19], HPLC–ELSD [20], and LC-MS/MS [21,22,23]. HPLC–ELSD was used to determine the main component and the organic impurities of D-mannitol in the work. Stability and homogeneity of the candidate CRM were also studied using the developed HPLC–ELSD method. A comprehensive investigation of the HPLC–ELSD parameters was carried out, where the indices included column, the column temperature, and mobile phase, as well as parameters of the detector that included the drift tube temperature, the carrier gas pressure, and the detector gain.

**Figure 2:**
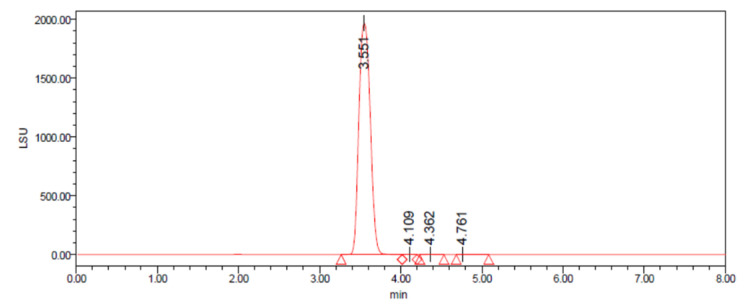
The HPLC–ELSD chromatogram for the candidate CRM.

**Figure 3:**
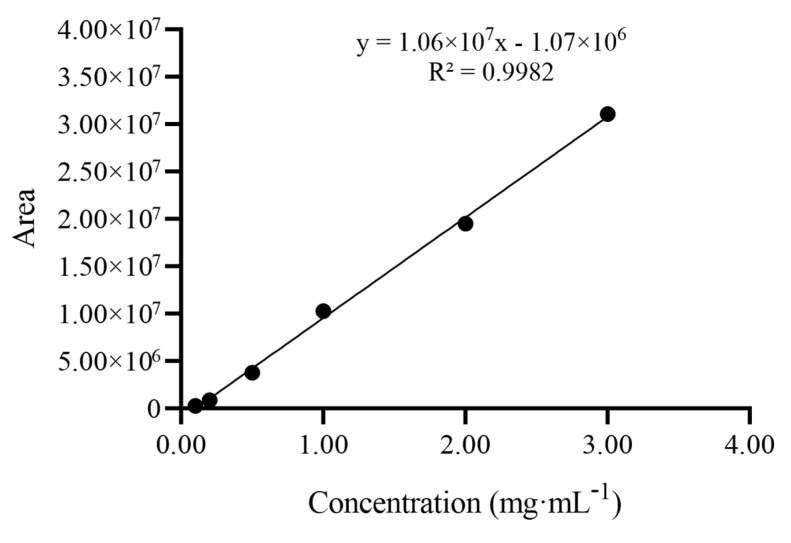
The calibration curve graph of the HPLC–ELSD method for determination of the candidate CRM.

After optimization, the main component for the candidate CRM was carried out on a Prevail Carbohydrate ES (250 mm × 4.6 mm, 5 μm) at 30 °C using water–acetonitrile (*v*/*v*, 60:40) as the mobile phase with the flow rate of 0.80 mL∙min^−1^. The drift tube temperature for the ELSD was set at 60 °C with the carrier gas pressure of 206.84 kPa and the detector gain of 50. The HPLC–ELSD chromatogram for the candidate CRM is shown in Figure 2. The working solutions with gradient concentrations (0.10, 0.20, 0.50, 1.00, 2.00, and 3.00 mg∙mL^−1^) were freshly prepared in water. The validation of HPLC–ELSD approach was also studied. As shown in Figure 3, the calibration curve was found to be linear for concentrations in the range of 0.10–3.00 mg∙mL^−1^, with linear regression analysis equation y = 1.06 × 10^7^x − 1.07 × 10^6^ (R ≥ 0.99). The limit of detection (LOD) and the limit of quantification (LOQ) were 5 μg∙mL^−1^ and 10 μg∙mL^−1^, respectively. In conclusion, the optimized HPLC–ELSD method was reproducible and accurate and can be used to determine the organic purity in D-mannitol.

### 2.3. Homogeneity Test

The results of homogeneity test are listed Table S2. As can be seen from Table S2, the calculated *F* value was less than the critical *F* value, indicating that the differences within and between bottles were not significant. According to ISO 17034 and ISO Guide 35 [9,10], the homogeneity of the candidate CRM was acceptable.

### 2.4. Stability Studies

A compound may degrade in some conditions, which causes the main content and the impurities to alter. Therefore, it is necessary to monitor the stability of the compound over time. The short-term stability and long-term stability were investigated using classical design stability experiments. For stability tests of the candidate CRM, short-term (transportation) stability at 50 °C and long-term (storage) stability at room temperature were investigated for 48 months and 28 days, respectively. The data were estimated using regression analysis. The statistical analyses of all the stability investigations were carried out to study if the apparent content degraded the candidate CRM during storage.

According to the results, the estimated slopes (*b*_1_) for the short-term stability and long-term stability were both below *t*_(0.95,3)_**·***s*(*b*_1_), as listed in Table S3. This indicated that the candidate CRM remained stable for up to 48 months under room temperature conditions and 28 days at 50 °C. To evaluate its expiry date, the long-term stability of the candidate CRM stored at room temperature is still to be monitored.

### 2.5. Mass Balance Approach

The purity (*P_MB_*, %) of the candidate CRM determined by the MB approach was calculated using Equation (1) [24]:

*P_MB_* = *P*_0_(1 − *X_V_* − *X_W_* − *X_NV_*), (1)

where *P_MB_* and *P*_0_ denotes the purity and HPLC purity *t* (%) of the candidate CRM, separately; *X_V_*, *X_W_*, and *X_NV_* denote the contents of volatile impurities (%), moisture (%), and nonvolatile impurities (%) of the candidate CRM, respectively.

Before impurity determination, different concentrations of the candidate CRM in water solutions were prepared to check the ELSD response of the main component and detectable impurities based on the developed HPLC–ELSD approach. The solutions with different concentrations of D-mannitol (0.10–3.00 mg∙mL^−1^) were measured. The results indicate that a solution of D-mannitol with a high concentration can ensure a sufficient ELSD response of impurities so that the purity of the candidate CRM can be accurately obtained. When the concentration of the candidate CRM exceeded 0.50 mg∙mL^−1^, the content of the main component and the impurities remained essentially constant, whereas when the concentration of the candidate CRM exceeded 3.00 mg∙mL^−1^, the ELSD response of the main component was overloaded. Therefore, 2.00 mg∙mL^−1^ was determined as the certified concentration to determine the main component for the candidate CRM.

Six bottles were selected from the candidate CRM, and three subsamples per bottle were prepared as solutions with the certified concentration. Then, the above solutions were analyzed using the above-described HPLC–ELSD approach in triplicate. The results of the main component of the candidate CRM were calculated using an area normalization method. The main component was 99.91% with the standard deviation (SD) of 0.01%, as listed in Table 1. The moisture content of the candidate CRM was 0.036% with an SD of 0.002%, as measured by Karl Fischer titration. The volatile impurities in the candidate CRM were determined by headspace GC–FID methods. As can be seen from Figure S2, no solvent was detected in the candidate CRM; therefore, the content of the volatile impurities could be ignored. The nonvolatile impurities determined by the ICP–MS were much less than 0.01%, which can also be ignored. According to Equation (1), *P_MB_* was 99.89% with an SD of 0.01% determined using the MB method.

### 2.6. Quantitative NMR Approach

The purity determined by the *q*NMR method was as follows [24,25]:

*P_x_* = *I_x_*/*I_std_*·*N_std_*/*N_x_*·*M_x_*/*M_std_*·*m_std_*/*m_x_*·*P_std_*. (2)

Here, *P* denotes the purity of the sample; *I* indicates the integrated signal areas of the sample; *N* represents the spin numbers of the sample; *M* denotes the molecular mass of the sample; *m* represents the mass of the sample. The subscripts *x* and *std* denote the analyte and IS, respectively.

The *q*NMR measurement, a suitable IS, and solvent should be firstly chosen. A primary CRM, as an IS, is required. Apart from the traceability to the SI unit, a suitable IS should have no signal peaks overlapped with the target analyte. Therefore, the IS selected here was benzoic acid CRM (GBW 06117), and the used solvent was DMSO-*d*_6_ in the measurement. The NMR parameters including excitation pulse angle, relaxation delay time d1, and scanning times, were optimized. The certified concentrations of the candidate CRM and the IS were optimized as 4.00 mg∙mL^−1^ and 2.00 mg∙mL^−1^, respectively. Six specimens were prepared to the solutions with certified concentrations and analyzed under the optimized NMR parameters.

**Figure 4:**
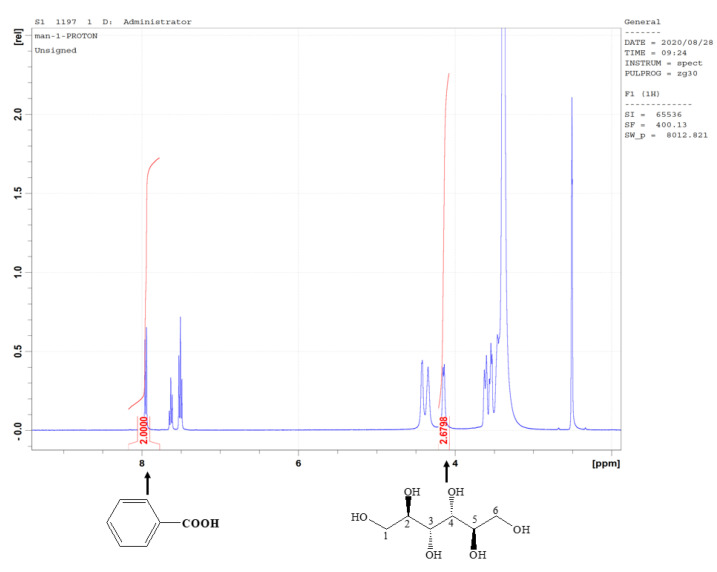
^1^H NMR spectra for the candidate CRM and benzoic acid.

The ^1^H NMR spectrum of the solution containing the candidate CRM and IS is shown in Figure 3. In the *q*NMR method, the ^1^H peaks selected as the quantitative peaks must be neither overlapping nor underlying impurity peaks. As shown in Figure 4, the ^1^H peaks in the OH-3 and OH-4 group (δ 4.14 ppm) of the candidate CRM and Ar–H (δ = 8.05 ppm) of the IS, are well resolved from other peaks and could be integrated properly. For this reason, these hydrogen peaks were chosen for quantification. The purity $P_{qNMR}$ of the candidate CRM was 99.90%, with an SD of 0.02% according to Equation (2).

### 2.7. Purity Certification

The purity of the candidate CRM, determined using the MB and *q*NMR methods, was cross-checked via a *t* test and an *F* test. The value of the *F*_test_ was less than *F*_0.05(5,5)_, which indicated that the two methods have equal accuracy. But *t*_test_ was greater than *t*_0.05(2,10)_, which indicated that there is a significant difference between the two methods. Thus, the certified purity P is 99.9% (Table 1), taking the average value of the MB and *q*NMR methods. The difference between the purity determined by the two methods is a part of the uncertainty of the certified value of the candidate CRM.

### 2.8. Uncertainty Estimation

According to ISO Guide 35 [10], the combined relative uncertainty ($u_{CRM-rel}$) of a CRM comprises the results of the homogeneity test $u_{bb-rel}$ stability studies (including short-term stability study ($u_{sst-rel}$) and long-term stability study ($u_{lst-rel}$)), and characterization ($u_{char-rel}$), that can be calculated as follows:

(3) $$u_{CRM-rel}=\sqrt{u_{char-rel}^{2}+u_{bb-rel}^{2}+u_{lst-rel}^{2}+u_{sst-rel}^{2}}.$$

#### 2.8.1. Uncertainty of the Mass Balance Approach

The combined uncertainty $u_{MB-rel}(P)$ of the MB method can be calculated using Equation (4) according to JJF 1855-2020 [11]:

(4) $$u_{MB-rel}(P)=\sqrt{{\left[u_{rel}(P_{0})\right]}^{2}+\frac{{\left[u\left(X_{V}\right)\right]}^{2}+{\left[u\left(X_{W}\right)\right]}^{2}+{\left[u\left(X_{NV}\right)\right]}^{2}}{{\left(1-X_{V}{-X}_{W}-X_{NV}\right)}^{2}}}$$

where *P*_0_ denotes the main component’s content; $u_{rel}(P_{0})$ indicates the relative uncertainty determined by the HPLC–ELSD method; *X_W_* and *u*(*X_W_*) represent the content and the uncertainty for moisture, respectively; *X_V_* and *u*(*X_V_*) represent the content and the uncertainty for volatile impurities, respectively; *X_NV_* and *u*(*X_NV_*) represent the content and the uncertainty for nonvolatile impurities, respectively, and nonvolatile impurities, respectively. Among them, $u_{rel}(P_{0})$ comprised *u_rel_*_,1_, *u_rel_*_,2_, and *u_rel_*_,3_, as calculated by Equation (5):

(5) $$u_{rel}(P_{0})=\sqrt{{u_{rel,1}}^{2}+{{{u_{rel,2}}^{2}{{+u}_{rel,3}}^{2}}_{}}^{}}$$

Here, *u_rel_*_,1_ relates to the relative SD of measurements, *u_rel_*_,2_ is correlated with the HPLC–ELSD response linearity, and *u_rel_*_,3_ is linked to the LOD of the HPLC–ELSD [11].

In Equation (5), $u_{rel,1}$ was calculated as $u_{rel,1}$ = 0.01%$/\sqrt{n}$ = 0.01%/$\sqrt{6}$ = 0.004%. Here, 0.01% is the relative SD (RSD) of six replicate measurements, as presented in Table 1.

Additionally, *u_rel_*_,2_ was estimated from the response linearity of the HPLC–ELSD method. The certified concentration of the candidate CRM was 2.00 mg/mL, which falls within the linear range of the method (0.05–3.00 mg/mL); therefore, *u_rel_*_,2_ could be negligible.

Moreover, *u_rel_*_,3_ was calculated from the LOD of the HPLC–ELSD method. The calculation equation was *u_rel_*_,3_ = LOD/*c* = 5 µg∙mL^−1^/2.00 mg∙mL^−1^ = 0.25% (11). Here, *c* is the certified concentration of D-mannitol candidate CRM.

Therefore, according to Equation (5), ${u_{rel}(P_{0})=\sqrt{{u_{rel,1}}^{2}+{u_{rel,3}}^{2}}}_{}=\sqrt{{\left(0.004\%\right)}^{2}+{\left(0.25\%\right)}^{2}}=0.26\%.$

The uncertainty from the moisture content $u\left(X_{W}\right)$ was calculated according to Equation (6) [9] and is shown in Table 2.

(6) $$u(X_{W})=X_{W}\sqrt{u_{rel,1}^{2}+{\left[\frac{u\left(m\right)}{m}\right]}^{2}{+\left[\frac{u\left(W\right)}{W}\right]}^{2}{+\left[\frac{u\left(f\right)}{f}\right]}^{2}}$$

Here, $X_{W}$ is the moisture of the candidate CRM; $u_{rel,1}^{}$ is the uncertainty for moisture repeat measurement; *m* and $\;u\left(m\right)$ are the weighed mass of the candidate CRM and the relevant certainty, respectively; *W* and $u\left(W\right)$ represent the weighed mass of the moisture in the candidate CRM and the relevant certainty, respectively. The value of f was determined using the moisture or temperature correction factor, and $u\left(f\right)$ is the standard uncertainty of f.

The RSD of the moisture measurements is 0.20%, as calculated from Table 1. Therefore, $u_{rel,1}^{}$ was 0.06%$/\sqrt{n}$ = 0.06%$/\sqrt{6}$ = 0.02% (*n* indicates the repeated number of the moisture measurements); $\frac{u\left(m\right)}{m}$ is the relative uncertainty from the weighed mass of the candidate CRM and was calculated as $\;\frac{u\left(m\right)}{m}$ = $\frac{0.01\;\mathrm{mg}}{200\;\mathrm{mg}*\sqrt{3}}\times 100\%=0.003\%$. Here, *m* was about 200 mg; $u\left(m\right)$ was 0.01 mg, as obtained by the accuracy of the used analytical balance (*d* = 0.01 mg); $\frac{u\left(W\right)}{W}$ is the relative uncertainty of the mass of the moisture and was calculated as $\;\frac{u\left(W\right)}{W}=\frac{10\;µg}{200\;\mathrm{mg}\times 0.036\%}\times 100\%=13.89\%$. Here, W was the product of the mass (m) of the candidate CRM and the content ($X_{W}$, 0.036%) of the moisture; $u\left(W\right)$ was 10 µg, taken from the resolution of the used moisture equipment. The moisture equipment used here was validated by moisture CRMs before the moisture measurement. The measured values were consistent with their certificate values; therefore, the relative uncertainty $\frac{u\left(f\right)}{f}$ caused by the correction coefficient of the moisture equipment could be negligible. $u\left(X_{W}\right)$ was calculated as $u\left(X_{W}\right)=X_{W}\sqrt{u_{rel,1}^{2}+{\left[\frac{u\left(m\right)}{m}\right]}^{2}{+\left[\frac{u\left(W\right)}{W}\right]}^{2}{+\left[\frac{u\left(f\right)}{f}\right]}^{2}}$ = 0.036% × $\sqrt{{\left[0.02\%\right]}^{2}+{\left[0.003\%\right]}^{2}{+\left[13.89\%\right]}^{2}}$ = 0.05%.

The contents of nonvolatile and volatile impurities were all far less than 0.001%; therefore, their related uncertainties can also be negligible according to Equation (1). According to Equation (4), $u_{MB-rel}(P)$ was calculated as follows: $u_{MB-rel}(P)=\sqrt{{\left[u_{rel}(P_{0})\right]}^{2}+\frac{{\left[u\left(X_{V}\right)\right]}^{2}+{\left[u\left(X_{W}\right)\right]}^{2}+{\left[u\left(X_{NV}\right)\right]}^{2}}{{\left(1-X_{V}-X_{W}-X_{NV}\right)}^{2}}}=\sqrt{{\left(0.26\%\right)}^{2}+\frac{{\left(0.05\%\right)}^{2}}{{\left(1-0.05\%\right)}^{2}}}$ = 0.27%.

#### 2.8.2. Uncertainty of the *q*NMR method

The combined relative uncertainty $u_{qNMR-rel}^{}$ of the *q*NMR method was calculated according to Equation (7) [24].

(7) u qNMR−rel=uIxIstdIxIstd2+uMxMx2+uMstdMstd2+umstdmstd2+umxmx2+uPstdkstd2

Here,uIxIstdIxIstd indicates the relative uncertainty of the quantitative peak area ratio; M and m indicate the molar mass and weighed mass of the compound, respectively; $u\left(M\right)$ and u(m) indicate the uncertainties of the foregoing two; $u\left(P_{std}\right)$ indicates the expanded relative uncertainty of IS; $k_{std}$ indicates the expanded factor of the uncertainty for IS. The subscripts *x* and std indicate the candidate CRM and IS, respectively.

Here, uIxIstdIxIstd can be calculated as follows: uIxIstdIxIstd=RSD/n=0.03%/6 = 0.02% (the RSD is 0.03% as listed in Table 1, *n* is the times of repeated measurements).

Additionally, $\frac{u\left(M_{x}\right)}{M_{x}}\;$ and $\frac{u\left(M_{std}\right)}{M_{std}}$ indicate the relative uncertainty of molar mass associated with the candidate CRM and IS, that were 0.005% and 0.003%, respectively, according to the reported calculation formula [24].

Moreover, $\frac{u\left(m_{std}\right)}{m_{std}}$ and $\frac{u\left(m_{x}\right)}{m_{x}}$ are the relative uncertainties that occurred from the weighed mass associated with D-mannitol and IS. Here,$\frac{u\left(m_{std}\right)}{m_{std}}$ consists of two parts: the relative uncertainty of the weighed mass by the used analytical balance $u_{rel,1}$ and the volumetric flask in the preparation of standard solutions $u_{rel,2}$ Additionally, $u_{rel,1}$ was calculated as follows: $u_{rel,1}=\frac{0.01\mathrm{mg}}{100.00\;\mathrm{mg}*\sqrt{3}}=0.006\%$. Here, 0.01 mg and 100.00 mg were the accuracy of the used analytical balance and the weighed mass of D-mannitol used for moisture measurement, respectively.

The relative uncertainty $u_{rel,2}$ generated by the calibration of the volumetric flask in the preparation of D-mannitol solutions consisted of two parts. The first part involved the relative uncertainty $u_{T}$ caused by changes in the volume of solvent in the volumetric flask due to temperature change, $u_{T}=\frac{0.00088\;\times \;(25\;-\;20)V}{\sqrt{3}}=\frac{0.00088\;\times \;5\;\times \;5}{\sqrt{3}}$ = $\frac{0.022}{\sqrt{3}}$, where *V* represents the volume of the volumetric flask, “25” indicates the temperature of preparation of the sample solution, “20” indicates the temperature of instrument calibration, and “0.00088” indicates the expansion coefficient of DMSO-*d*_6_. The second part involved the capacity tolerance of the volumetric flask, $u_{v}=\delta /\sqrt{3}=0.02/\sqrt{3}$, where “0.02” indicates the capacity tolerance of the volumetric flask used. Thus, the combined uncertainty can be expressed as $u\left(V\right)=\sqrt{u_{T}^{2}+u_{v}^{2}}=\sqrt{{\left(\frac{0.022}{\sqrt{3}}\right)}^{2}+{\left(\frac{0.02}{\sqrt{3}}\right)}^{2}}$ and the relative uncertainty can be expressed as $u_{rel,2}=\frac{u(V)}{V}=\frac{0.017}{5}\times 100\%=0.34\%$. Therefore, $\frac{u\left(m_{x}\right)}{m_{x}}$ was calculated as follows: $\frac{u\left(m_{x}\right)}{m_{x}}=\sqrt{{u_{rel,1}}^{2}+{u_{rel,2}}^{2}}=\sqrt{{\left(0.06\%\right)}^{2}+{\left(0.34\%\right)}^{2}}$ = 0.35%. By the same principle, $\frac{u\left(m_{std}\right)}{m_{std}}$ was 0.35%.

The relative uncertainty associated with the IS ($\frac{u\left(P_{std}\right)}{k_{std}}$) was 0.01%. Here, $u\left(P_{std}\right)$ and *k* were 0.02% and 2, respectively, as obtained from the certification of IS. The relative uncertainty of the IS was 0.02% (*k =* 2).

Based on the above results, $u_{qNMR-rel}^{}$ was calculated according to Equation (7):

$u_{qNMR-rel}^{}=\sqrt{{\left(0.02\%\right)}^{2}{+(0.35\%)}^{2}+{\left(0.35\%\right)}^{2}+{\left(0.01\%\right)}^{2}}=0.49\%$

Given that there is a significant difference between MS- and *q*NMR-based purity measurements, the relative uncertainty of characterization $u_{char-rel}$ was calculated using Equation (8):

(8) $$\begin{array}{cc} u_{char-rel} & =\sqrt{u_{MB-rel}^{2}+u_{qNMR-rel}^{2}+{\left(\frac{\left|P_{MB}-P_{qNMR}\right|}{2}\right)}^{2}}\\ & \;\;\;=\sqrt{{\left(0.27\%\right)}_{}^{2}+{\left(0.49\%\right)}_{}^{2}+{\left(\frac{\left|99.90\%-99.89\%\right|}{2}\right)}^{2}} \end{array}$$

where $P_{MB}$, $P_{qNMR}$ and $u_{MB-rel}^{}$, $u_{qNMR-rel}^{}$ separately denote the purities and the according relative uncertainties associated with the MB and *q*NMR approaches.

#### 2.8.3. Uncertainty of Homogeneity

The uncertainty of homogeneity ($u_{bb}$) resulted from the inhomogeneity within the bottles (${\mathrm{MS}}_{\mathrm{within}}$) and between the bottles (${\mathrm{MS}}_{\mathrm{among}}$). In this study, ${\mathrm{MS}}_{\mathrm{among}}$ > ${\mathrm{MS}}_{\mathrm{within}}$; therefore, $u_{bb}$ was calculated according to Equation (9) [8,24,26]:

(9) $$u_{bb}=\sqrt{\frac{{\mathrm{MS}}_{\mathrm{among}}-{\mathrm{MS}}_{\mathrm{within}}}{n}}=\sqrt{\frac{0.000132-0.000098}{3}}=0.01\%$$

The relative uncertainty $u_{bb-rel}$ was calculated according to $u_{bb-rel}=\frac{u_{bb}}{P_{}}=\frac{0.01\%}{99.9\%}\times 100\%=0.02\%$. Table 2 details the results.

#### 2.8.4. Uncertainty of Stability

The uncertainty of stability $u_{s}$ including the uncertainties of short-term stability ($u_{sts}$) and long-term stability ($u_{lst}$) is calculated as follows:

(10) $$u_{s}=s(b_{1})t$$

Therefore, $u_{lts}=0.000343\%\times 48=0.02\%,u_{sts}=0.000680\%\times 28=0.02\%$. The computational formulas for relative uncertainties of short-term stability $u_{sts-rel}$ and long-term stability $u_{lst-rel}$ are: $u_{lst-rel}=\frac{u_{lts}}{P}=\frac{0.02\%}{99.9\%}=0.03\%$ and $u_{sts-rel}=\frac{u_{sts}}{P}=\frac{0.02\%}{99.9\%}=0.03\%$, respectively. Here, *t* denotes the time of stability monitoring (months or days). The results are listed in Table 2.

#### 2.8.5. Combined and Expanded Uncertainties

According to Equation (3), the combined relative uncertainty $u_{CRM-rel}$ is 0.57%, which is formulated as follows: $u_{CRM-rel}=\sqrt{{\left(0.56\%\right)}_{}^{2}+{\left(0.02\%\right)}_{}^{2}+{\left(0.03\%\right)}_{}^{2}+{\left(0.03\%\right)}_{}^{2}}=0.57\%$. The uncertainty ($u_{CRM}$) of the purity for the candidate CRM is the product of $u_{CRM-rel}$ and the certified value P. The expanded relative uncertainty $U_{CRM-rel}$ was 1.1%, which is calculated as the product of the combined uncertainty $u_{CRM-rel}$ and *k* (*k* = 2). Table 2 details the results.

## 3. Materials and Methods

### 3.1. Chemicals and Materials

The raw D-mannitol used herein was purchased from Sigma-Aldrich (St. Louis, MO, USA). DMSO-*d*_6_ (>99.9%, containing 0.03% (*v*/*v*) TMS) was obtained from Tenglong (Qingdao, Shandong, China). Water Contents of Solid CRM (GBW 13518, 9.90 ± 0.20 mg.g^−1^), Water Contents of Sodium Tartrate Dihydrate CRM (GBW 13515, 156.3 ± 1.3 mg·g^−1^), Water Contents of Lactose Monohydrate CRM (GBW 13517, 50.07 ± 0.6 mg·g^−1^), and benzoic acid CRM (GBW 06117, 99.990 ± 0.009%), were purchased from NIM (National Institute of Metrology, Beijing, China). HPLC-grade methanol was obtained from Sigma-Aldrich (St. Louis). High-purity water was obtained through a Milli-Q water purification system (Millipore, Bedford, MA, USA).

The main component of the candidate CRM was measured on a Waters e2695 system that was equipped with an ELSD, a binary solvent manager, and an autosampler (Waters, Milford, MA, USA). The volatile impurities of the CRM were measured using a 7890B GC system (Agilent, Waltham, MA, USA) equipped with a headspace injector and an FID. The measurement of nonvolatile impurities and the water content were carried out on an iCAPQ ICP–MS (Thermo-Fisher, Waltham, MA, USA) and a model 831 Karl Fischer coulometer (Metrohm AG, Bleiche West, Switzerland), separately. NMR spectra were obtained using an AV400 spectrometer (Bruker, Karlsruhe, Baden-Württemberg, Germany). The Xevo G2 QTof (Waters) was used to detect the mass spectra. A Bruker Vertex 70 FT-IR spectrophotometer (Bruker) and a Thermo Fischer DXR spectrometer (Thermo, Waltham, MA, USA) were used for the analysis of infrared (IR) and Raman spectrum, respectively. A polarimeter (Anton Paar, Graz, Austria) was used to measure the optical rotation value. The samples used in the study were weighed on AL104 (Mettler-Toledo, Greifensee, Switzerland) (max = 110 g, *d* = 0.1 mg) and Quintix 35-1CN (Sartorius, Göttingen, Germany) (max = 32 g, *d* = 0.01 mg) analytical balances.

### 3.2. Methods

#### 3.2.1. Preparation of the Candidate CRM

A total amount of 100 g D-mannitol raw material was dried under decompression at 60 °C using a P_2_O_5_ desiccant based on dry mass measured until no further weight loss occurred. After hypobaric drying and homogeneous mixing, the samples were sealed in 2 mL brown glass bottles (each bottle contained 200 mg) and a batch of 400 bottles was stored at room temperature as the candidate CRM.

#### 3.2.2. Structural Analysis

##### High-Resolution Mass Spectra

HRMS of the candidate CRM was measured in a positive mode with electrospray ionization under the following conditions: scan range *m/z* 100–500, source temperature 120 °C, desolvation temperature 300 °C, capillary voltage 3500 V, sample cone voltage 40 V, cone gas (nitrogen) 15 L∙h^−1^, and desolvation gas (nitrogen) 300 L∙h^−1^. The HRMS spectrum was validated by comparing the detected mass with the theoretical mass.

##### Fourier-Transform Infrared (FT-IR) Spectra

FT-IR spectra were recorded using a Bruker Vertex-70 spectrophotometer. FT-IR spectra of the candidate CRM were collected via the KBr pellet technique. An appropriate amount of the candidate CRM was mixed with dried spectroscopic grade KBr powder (1:100) and the mixture was compressed into a pellet for FT-IR measurements. All the spectra were collected in the 4000–400 cm^−1^ range at 8 cm^−1^ resolution. The IR spectra were analyzed based on the structure of D-mannitol and compared with the standard spectra from the SBDS database and the reported literature [12,13].

##### Raman Spectra

Raman tests were performed on a Thermo Fischer DXR spectrometer, with a 532 nm laser and a spectral resolution of less than 2 cm^−1^. Raman spectra were analyzed based on the structure of D-mannitol and compared with the standard spectra from the SDBS database and the reported literature [12,14].

##### Optical Rotation Value

Optical rotation value was obtained on an AUTOPOL IV automatic polarimeter. A total of 25 mg of the candidate CRM was weighed accurately and placed in a 25 mL volumetric flask, then diluted with 8 mL of ammonium molybdate solution (0.1 g∙mL^−1^) and 4 mL of sulfuric acid solution (0.5 mol∙L^−1^), and was further diluted with water to prepare the candidate CRM solution of 1.00 mg·mL^−1^. The optical rotation value of the sample was measured using a polarimeter.

##### Nuclear Magnetic Resonance Spectra

All NMR spectra were recorded on a Bruker 400 MHz NMR spectrometer, operating at 400 MHz for ^1^ H, and 100 MHz for ^13^ C DMSO-*d*_6_ was used as the solvent. The candidate CRM solution with about 10 mg∙mL^−1^ was prepared and measured. The NMR spectra were analyzed based on the structure of D-mannitol and compared with the standard spectrum from SDBS database [12].

### 3.3. Characterization

#### 3.3.1. Development and Method Validation of HPLC–ELSD

The HPLC–ELSD condition for determining the main component of the candidate CRM was carried on a Prevail Carbohydrate ES (250 mm × 4.6 mm, 5 μm) at 30 °C. The mobile phase comprised water–acetonitrile (*v*/*v*, 60:40). The flow rate was 0.80 mL∙min^−1^. The drift tube temperature for the ELSD was set at 60 °C with the carrier gas pressure of 206.84 kPa and the detector gain of 50.

The working solutions with gradient concentrations (0.10, 0.20, 0.50, 1.00, 2.00, and 3.00 mg∙mL^−1^) were freshly prepared in water. The linearity of the developed method was determined by analyzing the six above-mentioned solutions. The resulting data were processed using Empower 2 (Waters, Milford, MA, USA).

The standard solution was diluted with water to different concentrations, and the resulting diluted solutions were injected into the HPLC–ELSD system for analysis. The LOD and LOQ values were estimated on the basis of the concentration capable of eliciting response three times and ten times of the average response of the baseline noise, respectively.

#### 3.3.2. Mass Balance Method

The main component for the candidate CRM was determined by the developed HPLC–ELSD method. The moisture, volatile impurities, and nonvolatile impurities of the candidate CRM were determined by Karl Fischer titration, GC–FID, and ICP–MS, respectively [24].

#### 3.3.3. Quantitative NMR Method

Benzoic acid CRM (GBW 06117) was used as the IS in this work. A total of 100 mg of the candidate CRM and 60 mg of IS were weighed accurately, and diluted with DMSO-*d*_6_ in 5 mL volumetric flasks. The sample solutions with different concentrations for *q*NMR measurement were prepared by mixing 100 µL of IS solution and different volumes of the candidate CRM solution. The ^1^H NMR spectra of the above solutions were measured to optimize the certified concentrations. The results indicated that the certified concentration for IS and the candidate CRM were 2.00 mg∙/mL^−1^ and 4.00 mg∙mL^−1^, respectively. Then, D-mannitol (20 mg) and IS (10 mg) were weighed accurately, and diluted with DMSO-*d*_6_ in 5 mL volumetric flasks to prepare the solution with the certified concentration. Six specimens of the solution were analyzed, and the purity was calculated using Equation (2).

The *q*NMR was performed on Bruker Avance III 400 MHz spectrometer, which was equipped with a pulsed field gradient probe at a temperature of 293.4 K. Measurement conditions were systematically optimized. The optimized parameters were as follows: temperature of probe 25.0 °C, size of probe 5 mm, angle of excitation pulse 45°, spectral data points 16 K, time-domain points 32 K, relaxing times 30 s, pulse intervals 4.15 µs, and scan times 32.

### 3.4. Homogeneity Test

According to JJF 1855–2020 [11], 15 bottles were randomly selected from the produced batch of candidate CRM for the homogeneity study. Three portions from each bottle were extracted and measured three times each using the established HPLC–ELSD method under the same conditions. Measurement of these 45 replicate samples was accomplished in a random order to avoid any possible trends regarding the measurement sequence. According to ISO Guide 35 [10], the data of the homogeneity study were evaluated using one-way analysis of variance (ANOVA).

### 3.5. Stability Study

A classical stability study approach was applied to investigate the stability of the prepared CRM and analytes under the given storage and transport conditions. For short-term stability, 15 randomly selected bottles of the candidate CRM samples were exposed to a temperature of 50 °C (simulation of possible transportation conditions) for predetermined durations of 1, 4, 7, 14, and 28 days, and for long-term stability, three bottles of the candidate CRM samples were randomly selected from the candidate CRM samples stored at room temperature for 1, 3, 6, 9, 12, 24, 36, and 48 months. Three subsamples of each bottle were analyzed in triplicate using the established HPLC–ELSD method at each specified time for the short-term and long-term stabilities. The stability data were assessed using regression analysis.

## 4. Conclusions

The D-mannitol CRM (GBW(E) 100681) was comprehensively investigated with SI traceable purity assessment for the first time. The structural identity of D-mannitol was confirmed by IR, HRMS, and NMR. The certified purity was 99.9% ± 1.1%, as determined using MB and *q*NMR methods. In the MB method, the moisture, the nonvolatile impurities, and volatile impurities of the candidate CRM were measured using Karl Fischer titration, ICP–MS, and the headspace GC–FID technique, respectively. The D-mannitol CRM in this study was found to be homogeneous, as inspected by HPLC–ELSD. The D-mannitol CRM was found to be stable at 50 °C for 28 days and for at least 48 months at room temperature. It has high purity, exhibiting stability and homogeneity, which could help improve the accuracy and traceability of laboratory results.

## Figures and Tables

**Table 1:** Determination results for the candidate CRM.

Number	HPLC–ELSD (%)	Moisture (%)	Volatile Impurities (%)	Nonvolatile Impurities (%)	Determination Results	
					MB (%)	*q*NMR (%)
1	99.91	0.0357	<0.01	<0.01	99.87	99.90
2	99.92	0.0341	<0.01	<0.01	99.89	99.88
3	99.92	0.0357	<0.01	<0.01	99.88	99.91
4	99.91	0.0342	<0.01	<0.01	99.88	99.89
5	99.91	0.0396	<0.01	<0.01	99.87	99.89
6	99.92	0.0368	<0.01	<0.01	99.88	99.90
Mean	99.91	0.0360	-	-	99.89	99.90
SD	0.01	0.0020	-	-	0.01	0.02
$t_{calculate}$	3.162					
$t_{0.5,10}$	2.228					
Conclusion	$t_{calculate}$ < $t_{0.5,10}$ the means are coincident					
$F_{calculate}$	3.66					
$F_{0.5(5,5)}$	5.05					
Conclusion	$F_{calculate}$ < $F_{0.5(5,5)}$, the standard deviations are coincident					

**Table 2:** The uncertainties of the candidate CRM.

Uncertainties	Sources	Results (%)
$u_{bb-rel}^{}$	Inhomogeneity among the bottles and within bottles	0.03
$u_{lts-rel}^{}$	Instability at long-term storage condition	0.03
$u_{sts-rel}^{}$	Instability at short-term transportation condition	0.03
$u_{rel}(P_{qNMR})$	Purity characterization by qNMR	0.49
$u_{rel}(P_{MB})$	Purity characterization by MB	0.27
$u_{char-rel}$	Combined $u_{rel}(P_{qNMR})$ and $u_{rel}(P_{MB})$	0.56
$u_{CRM-rel}$	Combined $u_{bb-rel}^{},\;u_{lts-rel}^{},\;u_{sts-rel}^{},$ and $u_{char-rel}$	0.57
$U_{CRM-rel}$	Relative expanded uncertainty, $u_{CRM-rel}$ × *k* (*k* = 2)	1.1
$U_{CRM}$	Expanded uncertainty, $U_{CRM-rel}$ × *P*	1.1

## Data Availability

All data supporting the conclusions of this article are included in this article.

## Supplementary Materials

The following supporting information can be downloaded at: https://www.mdpi.com/article/10.3390/molecules28196794/s1, Figure S1: Chemical structure of the D-Mannitol candidate CRM; Figure S2: The head-space GC-FID spectra of the samples: (a) blank; (b) ethanol; (c) the candidate CRM; Table S1: The NMR data of the D-mannitol candidate CRM (DMSO-d_6_); Table S2 The results of homogeneity for the D-mannitol candidate CRM; Table S3: Stability results of the D-Mannitol candidate CRM.

## Sample Availability

Samples of the compound are available from the authors.
