# Supplementary material for: Development of a Pure Certified Reference Material of D-Mannitol
Source: Molecules. 2023 Sep 25;28(19):6794. doi: 10.3390/molecules28196794 (PMC10574156; doi:10.3390/molecules28196794)
Supplement: Supplementary file 1 [file molecules-28-06794-s001.zip › Table S1.pdf]

Table S1 The NMR data of the D-mannitol candidate CRM (DMSO-d_6)

Assignment	δ_H (ppm, 400 MHz)	δ_C (ppm, 100 MHz)
1-OH，6-OH	4.335(t,2H) J=3.4Hz	
3-OH，4-OH	4.140(d,2H) J=3.4Hz	
1，6	3.587～3.636(m,2H), 3.377～3.352(m,2H	64.33
2，5	3.523～3.562(m,2H), 4.411(br,2H)	71.77
3，4	3.390～3.483(m,2H)	70.12
