# Supplementary material for: Development of a Pure Certified Reference Material of D-Mannitol
Source: Molecules. 2023 Sep 25;28(19):6794. doi: 10.3390/molecules28196794 (PMC10574156; doi:10.3390/molecules28196794)
Supplement: Supplementary file 1 [file molecules-28-06794-s001.zip › Table S2.pdf]

Table S2 The results of homogeneity for the D-mannitol candidate CRM

Number	1	2	3	Means
i=1	99.92	99.90	99.92	99.91
i=2	99.91	99.92	99.93	99.92
i=3	99.92	99.93	99.91	99.92
i=4	99.90	99.93	99.92	99.92
i=5	99.93	99.92	99.92	99.92
i=6	99.93	99.91	99.91	99.92
i=7	99.93	99.93	99.94	99.93
i=8	99.90	99.92	99.90	99.91
i=9	99.91	99.91	99.91	99.91
i=10	99.91	99.93	99.91	99.92
i=11	99.92	99.93	99.92	99.92
i=12	99.91	99.93	99.93	99.92
i=13	99.92	99.91	99.92	99.92
i=14	99.90	99.91	99.92	99.91
i=15	99.91	99.93	99.91	99.92
$F=\frac{MS_{among}}{MS_{within}}$		1.34		
$F_{0.05}$ (14, 30)		2.04		
Conclusion		$F<F_{0.05}$ (14，30)		
