# Supplementary material for: Development of a Pure Certified Reference Material of D-Mannitol
Source: Molecules. 2023 Sep 25;28(19):6794. doi: 10.3390/molecules28196794 (PMC10574156; doi:10.3390/molecules28196794)
Supplement: Supplementary file 1 [file molecules-28-06794-s001.zip › Table S3.pdf]

Table S3 Stability results of the D-Mannitol candidate CRM

Stability	b_1	$s(b_1)$	Time	$t_{0.95,n-2}$	Conclusion
Long-term stability	0.000450	0.000343	48 months	2.365	$\|b_1\|<t\cdot s(b_1)$, stable
Short-term stability	-0.000749	0.000680	28 days	2.776	$\|b_1\|<t\cdot s(b_1)$, stable
