# Supplementary figures and images for: Development of a Pure Certified Reference Material of D-Mannitol
Source: Molecules. 2023 Sep 25;28(19):6794. doi: 10.3390/molecules28196794 (PMC10574156; doi:10.3390/molecules28196794)

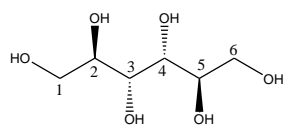

Figure S1. Chemical structure of the D-Mannitol candidate CRM

Supplement: Supplementary file 1 [file molecules-28-06794-s001.zip › Figure S1.pdf]

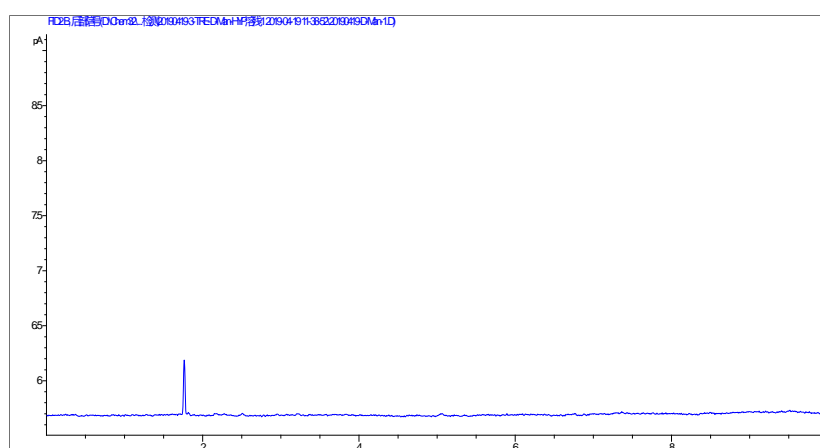

(c)

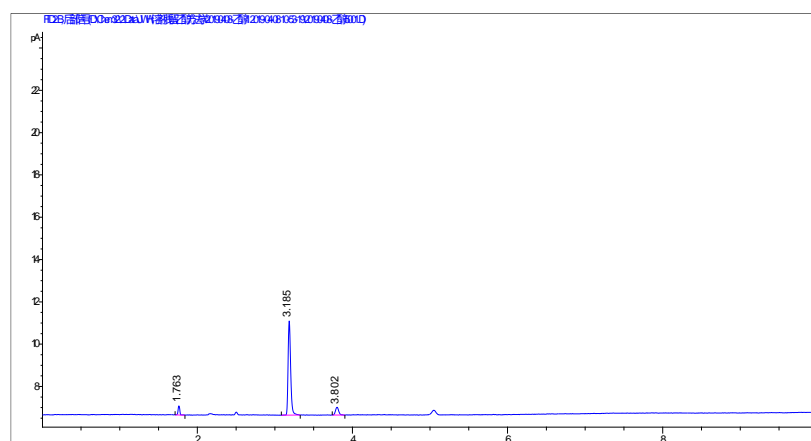

(b)

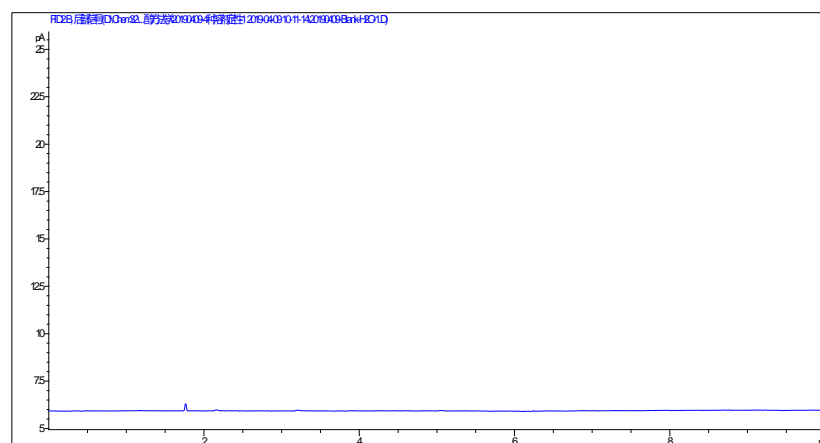

(a)

Figure S2 The head-space GC-FID spectra of the samples: (a)blank;  
(b)ethanol; (c) the candidate CRM

Supplement: Supplementary file 1 [file molecules-28-06794-s001.zip › Figure S2.pdf]
